# Lysophosphatidic Acid Induces MDA-MB-231 Breast Cancer Cells Migration through Activation of PI3K/PAK1/ERK Signaling

**DOI:** 10.1371/journal.pone.0015940

**Published:** 2010-12-30

**Authors:** Jun Du, Chongqi Sun, Zhenzhen Hu, Yu Yang, Yichao Zhu, Datong Zheng, Luo Gu, Xiang Lu

**Affiliations:** 1 Department of Physiology, Nanjing Medical University, Nanjing, China; 2 Kangda College, Nanjing Medical University, Nanjing, China; 3 Cancer Center, Nanjing Medical University, Nanjing, China; 4 The Second Affiliated Hospital of Nanjing Medical University, Nanjing, China; Florida International University, United States of America

## Abstract

**Background:**

Enhanced motility of cancer cells is a critical step in promoting tumor metastasis. Lysophosphatidic acid (LPA), representing the major mitogenic activity in serum, stimulates migration in various types of cancer cells. However, the underlying signaling mechanisms for LPA-induced motility of cancer cells remain to be elucidated.

**Methodology/Principal Findings:**

In this study, we found that LPA dose-dependently stimulated migration of MDA-MB-231 breast cancer cells, with 10 µM being the most effective. LPA also increased ERK activity and the MEK inhibitor U0126 could block LPA-induced ERK activity and cell migration. In addition, LPA induced PAK1 activation while ERK activation and cell migration were inhibited by ectopic expression of an inactive mutant form of PAK1 in MDA-MB-231 cells. Furthermore, LPA increased PI3K activity, and the PI3K inhibitor LY294002 inhibited both LPA-induced PAK1/ERK activation and cell migration. Moreover, in the breast cancer cell, LPA treatment resulted in remarkable production of reactive oxygen species (ROS), while LPA-induced ROS generation, PI3K/PAK1/ERK activation and cell migration could be inhibited by N-acetyl-L-Cysteine, a scavenger of ROS.

**Conclusions/Significance:**

Taken together, this study identifies a PI3K/PAK1/ERK signaling pathway for LPA-stimulated breast cancer cell migration. These data also suggest that ROS generation plays an essential role in the activation of LPA-stimulated PI3K/PAK1/ERK signaling and breast cancer cell migration. These findings may provide a basis for designing future therapeutic strategy for blocking breast cancer metastasis.

## Introduction

Lysophosphatidic acid (LPA), a naturally occurring phospholipids, represents the major mitogenic activity in serum. Platelet-derived LPA is an important mediator in wound healing and tissue regeneration [1], [2]. It is noteworthy that LPA also acts as a chemoattractant in promoting motility of various types of human cancer cell. More recently, LPA and its receptor have been demonstrated to be involved in promoting breast cancer metastasis to bone [3], [4]. Importantly, specific inhibition of LPA receptors abolishes the migration of cancer cell response to malignant ascites which contain LPA [5]. However, the molecular mechanisms underlying the effect of LPA on tumor cell migration are not completely understood to date.

Previous studies suggest that breast cancer progression is mediated by Autotaxin (ATX)–LPA signaling axis [6]. ATX and LPA1–3 receptors are expressed in mammary glands and can exert multiple effects under physiological conditions [7], [8]. LPA is produced from lysophosphatidylcholine (LPC) by ATX and acts on the EDG-family LPA receptors, LPA1, LPA2 and LPA3 [9]. As an important pathway promoting cell survival, the ATX–LPA signalling axis may initiate tumorigenesis in breast by making cells susceptible to other genetic mutations, leading to the accumulation of several aberrant signaling pathways. Indeed, each of these components of the ATX–LPA signaling axis sufficiently induces tumorigenesis through the upregulation of many signaling pathways, including signaling pathways via PI3K, MAPK, Wnt/β-catenin and estrogen receptor [7], [10], [11]. In addition, marked increase in the production of several cytokines by LPA further advance the disease progression by promoting local inflammation and angiogenesis [12]. The effects of LPA on cytokine production and blood vessel formation may contribute to the metastasis of breast tumors to other organs such as bone. However, the signaling mechanisms underlying the effect of LPA on breast cancer cell migration remains unelucidated.

P21-activated kinase 1 (PAK1), a main downstream effector of Rac1 and Cdc42, plays a pivotal role in signal transduction and cellular regulation of morphogenesis, survival, proliferation and motility [13], [14], [15], [16]. Emerging evidence has suggested that PAK1 is required for the progression and metastasis of cancer cells by mediating growth factor-induced motility and invasiveness [17], [18]. A large body of evidence indicates that PAK1 can be activated by some stress stimuli such as LPA [19]. In a study of human melanoma cells, the activation of PI3K-PAK1 signaling pathway is associated with focal adhesion kinase (FAK) phosphorylation and cell motility induced by LPA [20]. The activation of PAK1 during LPA stimulation evokes cellular ruffling activity in three-dimensional collagen matrix cultures [21]. In addition, LPA has been identified as a potent modulator of extracellular-signal-regulated kinase (ERK) activity [22]. ERK, a member of the mitogen-activated protein kinase (MAPK) family, is reported to be associated with lamellipodial dynamics [23] and chemotactic migration via a ROS-dependent way [24], [25], [26]. A recent study has shown that PAK1 can phosphorylate and activate both MEK1 [27] and Raf [28], which are upstream activators of ERK. Thus, it is worthwhile to explore whether the PAK1 and ERK signaling pathway is involved in LPA-induced cancer cell migration. In the present study, we investigated the signaling mechanisms underlying the effect of LPA on breast cancer cell migration. The PI3K/PAK1/ERK signaling pathway for LPA-stimulated breast cancer cell migration is identified and LPA is found to stimulate ROS generation in breast cancer cells.

## Results

### LPA stimulates breast cancer cell migration *in vitro*

**Figure 1:**
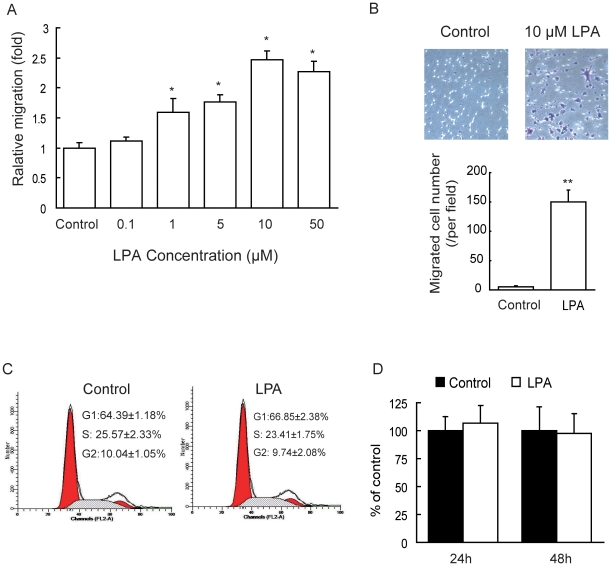
Effect of LPA on MDA-MB-231 cell migration. (A) Relative cell migration rate was determined using wound closure assay in MDA-MB-231 cells incubated in the absence (control) or presence of 0.1, 1, 5, 10 or 50 µM LPA for 18 h. (B) The cell migration was assessed by the transwell migration assay in MDA-MB-231 cells incubated in the absence (control) or presence of 10 µM LPA for 3 h. Representative micrographs of the migrated cells stained with crystal violet (upper panel) and the migrated cell number per field (lower panel). (C) MDA-MB-231 cells were cultured in the absence (control) or presence of 10 µM LPA for 24 h and cell cycle was analyzed by flow cytometry. (D) MDA-MB-231 cells were cultured in the absence (control) or presence of 10 µM LPA for 24 or 48 h and cell proliferation was analyzed by MTT assay. Each value is the mean ± SD of 6 independent determinations. *: *P*<0.05, **: *P*<0.01 in the cultures with LPA relative to the cultures without LPA.

To assess the effect of LPA on breast cancer cell migration, MDA-MB-231 cells were treated with various concentrations of LPA, and the migration rate of cells was measured by wound closure assay after the LPA treatment. Similar to the findings of Li et al [29], our results showed that the migration rate was increased by 111.94±5.60, 159.70±22.16, 176.12±12.31, 246.27±15.60 and 226.87±17.61% in the treatment of cells with 0.1, 1, 5, 10, and 50 µM LPA, respectively, as compared to control (Fig. 1A). Cell migration was also assessed by transwell migration assay. MDA-MB-231 cells were treated with 10 µM LPA, the migrated cell number was increased 30-folds in LPA treated cultures compared to control (Fig. 1B). To determine whether LPA-induced cell migration is associated with increased cell proliferation, MDA-MB-231 cells were treated with 10 µM LPA for 24 h, and the proliferation of the cells was examined by cell cycle analysis. The results revealed that the percentages of cells in the S and G_2_ phase were not altered significantly in LPA treated cells compared to the control cells (Fig. 1C). Our MTT assays also showed that treatment with 10 µM LPA for 24 and 48 h did not noticeably increase the proliferation of MDA-MB-231 cells (Fig. 1D).

### LPA stimulates cell migration by activating ERK

**Figure 2:**
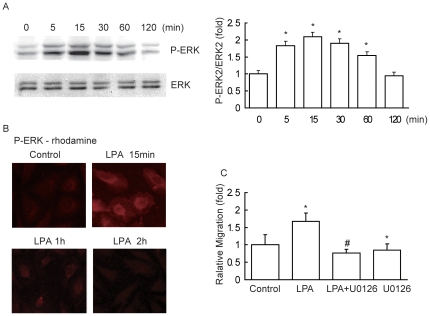
LPA-stimulated cell migration occurred by activation of ERK. (A) Effect of LPA stimulation on phosphorylation of ERK at Thr202/Tyr204. Confluent, serum-starved cells were treated with 10 µM LPA for the indicated periods. After stimulation, cells were analyzed for P-ERK or total ERK as described under “Experimental Procedures.” Data are presented as mean±SD of 3 determinations. (B) Representative micrographs of cells treated with 10 µM LPA for 0 (control), 15, 60 and 120 min and stained immunofluorescence for P-ERK. (C) Effect of U0126 on LPA-stimulated cell migration. Cells were incubated for 18 h in the absence or presence of 10 µM U0126, with 10 µM LPA or without LPA. Data are presented as mean±SD of 8 independent determinations. *: *P*<0.05 in the cultures with LPA relative to the cultures without LPA. #:*P*<0.05 in the cultures with LPA and U0126 relative to the cultures with LPA alone.

To explore the mechanism whereby LPA stimulates breast cancer cell migration, we first examined endogenous ERK activation after LPA treatment. LPA treatment resulted in a time-dependent increase in ERK activity, as determined by Western blotting with an antibody against the phosphorylated form of ERK (Fig. 2A). ERK activity was increased 5 to 60 min after 10 µM LPA treatment, with a maximum at 15–30 min. ERK activity was returned to basal levels at 120 min. As a control, the levels of total ERK were constant at all these time points (Fig. 2A). We also treated cells with different doses of LPA (0.1–10 µM) for 15 min, and found that LPA dose-dependently activated ERK in MDA-MB-231 cells (Figure S1).

Since ERK regulates cell migration not only depending on its phosphorylation status but also on its redistribution to the nucleus and plasma membrane [30], we also examined P-ERK localization in cultured cells after LPA treatment. Immunofluorescence staining revealed that P-ERK was weak and localized in the cytoplasm of serum-starved cells. However, P-ERK abundance was obviously increased in the cytoplasm, and large amounts of P-ERK could also be found in the nucleus and periphery of the cells at 15 min after exposure to LPA; The P-ERK was still detectable in both nucleus and cytoplasm at 1 h after the addition of LPA and was diminished in the nucleus and cytoplasm 2 h later (Fig. 2B).

To determine whether this nucleus translocation of P-ERK was associated with an increase of ERK transcriptional activity, we chose to analyze the level of one of these genes, MMP-9, which is frequently used as a primary mediator of cell migration and invasion [31]. We first quantified the levels of MMP-9 mRNA during LPA stimulation with RT-PCR. As shown in Figure S2, MMP-9 mRNA increased significantly 1 h after LPA stimulation, then decreased to near baseline levels approximately 2 h later. Pretreatment with LY294002 or U0126 inhibited LPA-stimulated MMP-9 induction in MDA-MB-231 cells. However, VEGF transcript levels in MDA-MB-231 cells were not altered in the presence of LPA (Figure S2). These results suggest that nucleus translocation of P-ERK might be required for the transcription of some mediators of migration such as MMP-9 induction.

To determine whether LPA stimulated migration of breast cancer cells depended on ERK activation, we investigated the effect of MEK inhibitor U0126 on cell migration using wound closure assay. Treatment of cells with 10 µM LPA increased the migration of MDA-MB-231 cells by 2.5 folds when compared with control cells. Pretreatment with U0126 not only eliminated LPA-stimulated migration, but also reduced cell migration in the absence of LPA treatment (Fig. 2C). These results indicate that the activation of ERK is essential for both basal and LPA-stimulated breast cancer cell migration.

### PAK1 mediates LPA-induced ERK activation and cell migration

**Figure 3:**
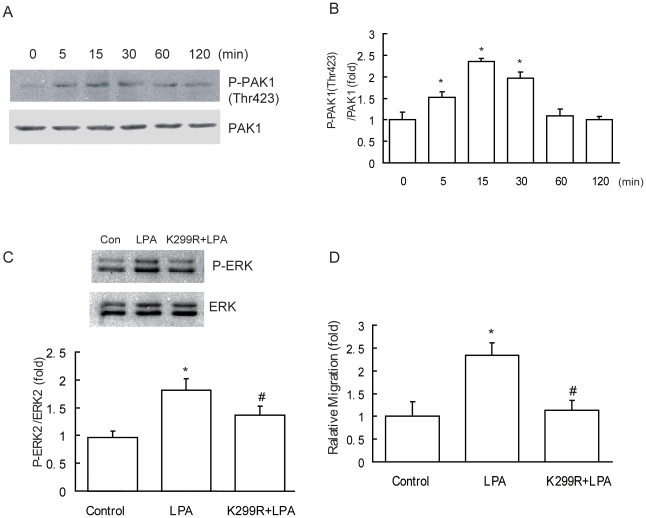
LPA activated ERK and enhanced cell migration mediated through PAK1. (A & B) Effect of LPA stimulation on phosphorylation of PAK1. Confluent, serum-starved cells were treated with 10 µM LPA for the indicated periods. LPA-stimulated phosphorylation of PAK1 at Thr423 was determined as described under “Experimental Procedures.” (C) LPA activated ERK dependent on PAK1. MDA-MB-231 cells transfected with either an empty vector or a PAK1 K299R expression vector were stimulated with 10 µM LPA for 15 min and ERK activity was examined. (D) Effect of the inactive mutant of PAK1 on LPA-stimulated migration. Cells transfected with empty vector or the PAK1 K299R expression vector were incubated with 10 µM LPA for 18 h and the cell migration rate was assessed by wound closure assay. Data are presented as mean±SD of 8 determinations. *: *P*<0.05 in the cultures with LPA relative to the cultures without LPA. #: *P*<0.05 in the cells transfected with the PAK1 K299R expression vector with LPA relative to the cells transfected with empty vector with LPA.

Previous reports have shown that growth factor-induced ERK activation is PAK1-dependent, and Thr 423, which resides in the activation loop of the catalytic domain, is a key intramolecular phosphorylatable residue for completing enzymatic activation of PAK1 [32]. Therefore, we examined whether PAK1 activation also occurred in our system. Western blotting analysis showed that the amount of phosphorylated PAK1 at Thr 423 was increased significantly from 5 to 30 min after LPA stimulation with maximal activation at 15 min. However, after 30 min it declined toward basal levels (Fig. 3A and B).

To determine whether LPA activates ERK via the PAK1 pathway, we overexpressed an inactive mutant of PAK1 in MDA-MB-231 cells and showed that it inhibited LPA-induced ERK phosphorylation. MDA-MB-231 cells transfected with either an empty vector or a PAK1 K299R expression vector were stimulated with 10 µM LPA for 15 min and ERK activity was examined. Following LPA stimulation, the activity of ERK was increased significantly in the control cells transfected with the empty vector, but it was abolished in the cells transfected with the PAK1 K299R expression vector (Fig. 3C).

To further determine whether LPA stimulates cancer cell migration in a PAK1-dependent manner, we investigated MDA-MB-231 cells migration using wound closure assay after transfecting these cells with the inactive mutant of PAK1 expression vector. 18 h after treatment with 10 µM LPA, the cell migration rate was increased significantly by 234.55±27.31% compared with control cells without LPA treatment. However, in cells transfected with the PAK1 K299R expression vector, such stimulatory effect of LPA on cell migration was eliminated (Fig. 3D). Consequently, the activation of PAK1 is essential for LPA-stimulated breast cancer cell migration.

### LPA induces PAK1/ERK activation and cell migration through PI3K pathway

**Figure 4:**
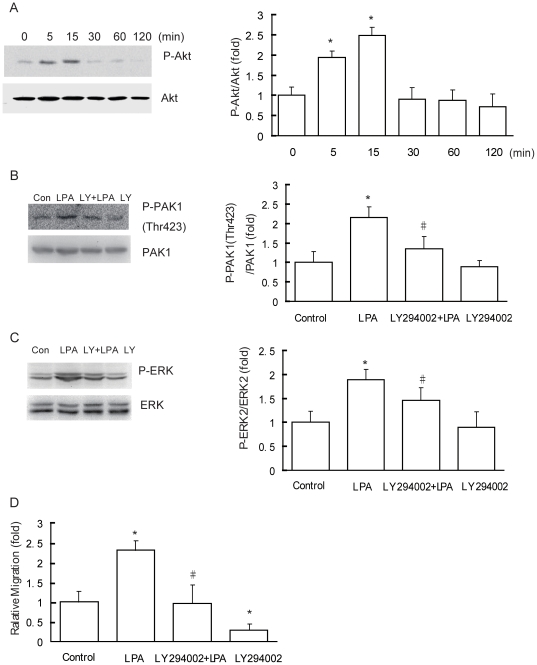
LPA activated PAK1/ERK and enhanced cell migration mediated through PI3K. (A) Effect of LPA stimulation on phosphorylation of Akt. Confluent, serum-starved cells were treated with 10 µM LPA for the indicated periods. LPA-stimulated phosphorylation of Akt was determined as described under “Experimental Procedures.” (B) LPA activation of PAK1 was dependent on PI3K. (C) LPA activation of ERK was dependent on PI3K. After treatment with 10 µM LY294002 for 30 min, cells were stimulated with 10 µM LPA for 15 min and then cells were analyzed by PAK1 (B) or ERK (C) assay as described under “Experimental Procedures.” Data are presented as mean±SD of 3 independent determinations. (D) Effect of PI3K inhibitor on LPA-stimulated cell migration. After pretreatment with 10 µM LY294002 for 30 min, cells were incubated with 10 µM LPA for 18 h and the cell migration rate was determined by wound closure assay. Data are presented as mean±SD of 8 independent determinations. *: *P*<0.05 in the cultures with LPA relative to the cultures without LPA. #:*P*<0.05 in the cultures with LPA and LY294002 relative to the cultures with LPA alone.

Previous report has shown that PI3K can activate both PAK1 and ERK in natural killer cells [33]. To determine whether PI3K is the upstream mediator of PAK1/ERK activation by LPA in our system, western blotting analysis of P-Akt (Ser473), a well accepted downstream target of PI3K, was used to determine the PI3K activity [34], [35]. The Results revealed a time-dependent increase in PI3K activity following LPA treatment. P-Akt was induced significantly from 5 to 15 min after LPA stimulation and maximal activation was at 5 min. P-Akt abundance returned to basal levels 15 min later (Fig. 4A).

To determine whether LPA-stimulated PAK1 and ERK activities are PI3K-dependent, we blocked PI3K activity by treating the cells with LY294002, a PI3K inhibitor, and examined PAK1 and ERK activities after stimulation with LPA. The results showed that pretreatment with 10 µM LY294002 largely inhibited LPA-induced PAK1 phosphorylation in comparison with control cells (Fig. 4B). ERK phosphorylation was also inhibited by LY294002 (Fig. 4C).

The effect of PI3K inhibitor on cell migration was also investigated using wound closure assay. Pretreatment with 10 µM LY294002 resulted in a remarkable inhibition of both basal and LPA-promoted cell migration (Fig. 4D). These results suggest that PI3K acts as the upstream effector of PAK1 and ERK in mediating LPA-stimulated breast cancer cell migration.

### ROS generation is required for LPA-induced PI3K/PAK/ERK activation and cell migration

**Figure 5:**
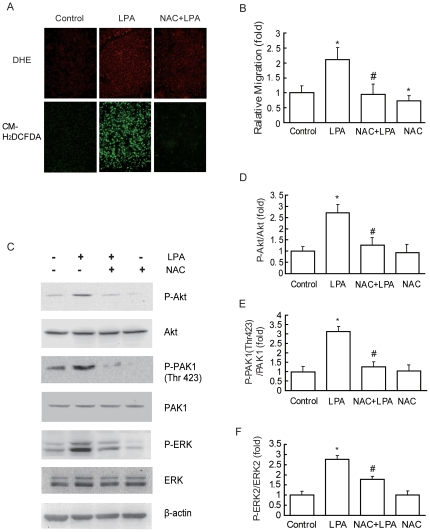
ROS is required for LPA-induced PI3K, PAK1 and ERK activation and cell migration. (A) Representative micrographs of MDA-MB-231 cells incubated without LPA (control, left panel) or with 10 µM LPA (middle panel) or with both 10 µM LPA and 2 mM NAC (right panel) and stained with DHE (upper panel) or with CM2-DCFHDA (lower panel). (B) Effect of ROS inhibitor on LPA-stimulated migration. After pretreatment with 2 mM NAC for 1 h, cells were incubated with 10 µM LPA for 18 h, the migration rate was determined by wound closure assay. (C) Effect of NAC on LPA-induced PI3K, PAK1 and ERK phosphorylation. After treatment with 2 mM NAC for 1 h, cells were stimulated with 10 µM LPA for 15 min and then cells were analyzed by Akt or PAK1 or ERK assay as described in “Experimental Procedures”. (D) Relative P-Akt expression levels. (E) Relative P-PAK1 (Thr423) expression levels. (F) Relative P-ERK expression levels. *: *P*<0.05 in the cultures with LPA relative to the cultures without LPA. #:*P*<0.05 in the cultures with LPA and NAC relative to the cultures with LPA alone.

ROS generation is frequently associated with an aggressive phenotype in human cancer [36]. We also examined the effect of LPA on ROS generation in cultured breast cancer cells. Superoxide anion and hydrogen peroxide (H_2_O_2_) production in cells were measured by fluorescent staining with dihydroethidium (DHE) and CM_2_-DCFHDA, respectively. The staining results revealed that superoxide anion and H_2_O_2_ abundance was weak in serum-starved cultured cells, but they were increased dramatically in 10 µM LPA-treated cells (Fig. 5A).

To investigate whether ROS generation leads to LPA-induced activation of the PI3K/PAK1/ERK signaling pathway and cell migration in cultured breast cancer cells, N-acetyl-L-cysteine (NAC), a known scavenger of ROS, was used and its effect on LPA-induced PI3K/PAK1/ERK activation was examined. Pretreatment with 2 mM NAC remarkably abolished superoxide anion and hydrogen peroxide (H_2_O_2_) production induced by LPA (Fig. 5A). Pretreatment with 2 mM NAC also largely inhibited LPA-induced PI3K, PAK1 and ERK activation (Fig. 5C). Furthermore, basal and LPA-stimulated cell migrations were blocked markedly by the pretreatment with NAC (Fig. 5B). These results indicate that LPA can stimulate ROS generation in breast cancer cells, and which may be an important mechanism for the stimulation of PI3K/PAK1/ERK signaling pathway and cell migration by LPA.

## Discussion

LPA and its receptors, LPA1 and LPA2, have been implicated in breast cancer [37]. In an in vivo model, the activation of LPA1 promotes breast cancer cell metastasis to the bone [4]. Similarly, in our system, breast cancer cell migration rate was accelerated after LPA treatment comparing with the control, but cell proliferation was not altered. Therefore, LPA directly activates the migration of breast cancer cells, which is a critical step for tumor metastasis. Based on this, the signaling mechanisms underlying the effect of LPA on promoting breast cancer cell migration were investigated.

A primary observation in the present study is that LPA induced activation of ERK in a time-dependent fashion in breast cancer cells. In addition, we observed that LPA not only enhanced the phosphorylation level of ERK in the cytoplasm and but also increased its redistribution from cytoplasm to nucleus and periphery of the cells. ERK is a known effector for cytoskeletal regulation and focal adhesion renewal. ERK activation promotes migration of various types of cell, including endothelial cells [38], [39], mesenchymal stem cells [40] and pancreatic cancer cells [41]. Previous study has shown that the activation of MAPK mediates LPA-stimulated preosteoblast migration [22], and is linked to the progression of breast cancer [42]. Our results found that when ERK signaling was blocked, LPA-stimulated cell migration was dramatically diminished. Therefore, our results suggest that ERK activation serves as a mediator of LPA-stimulated breast cancer cell migration.

We next examined the potential activators for ERK in our system. A previous study has demonstrated that the expression of a constitutively active form of PAK1 induces the rapid formation of lamellipodia, filopodia, and dorsal ruffles, as well as an increase in the reorganization of actin cytoskeleton and cell migration [43]. PAK1 has been reported to regulate the ERK pathway by direct interaction between PAK1 and Raf and MEK. PAK directly phosphorylates MEK1 on S298, which then induces MEK1 autophosphorylation on S218/222 and increases kinase activity toward ERK [44] and phosphorylates Raf on Ser338 [28], which is known as MEK activator. Our results show that LPA induces a time-dependent increase in PAK1 activity. Blocking PAK1 activity by ectopic expression of a kinase-dead PAK1 mutant significantly prevents LPA-induced ERK activation and cell migration. Previous study has shown that the inhibition of PAK1 activity down-regulates ERK signal transduction and reduces cell migration through affecting actin and adhesion dynamics [45]. Therefore, it may be reasonable to think that LPA-induced activation of ERK and enhanced cell migration is mediated through PAK1.

We then examined possible activators of PAK1 in our system. The activation of PI3K might affect the PAK1 signaling cascade. For example, the inhibition of PI3K activity blocks the activation of PAK1 and cell migration in response to heregulin [46]. Meanwhile, the association of PI3K with PAK1 has been reported to regulate PAK1 kinase activity and lead to cytoskeletal reorganization [47]. There has a good evidence suggesting a link from PI3K and Rac1, via PAK1, to ERK activation, which is essential for the regulation of cytotoxicity in natural killer cells [33]. Consistent with these reports, our results reveal that LPA triggers a rapid stimulation of PI3K activity. The selective PI3K inhibitor LY294002 effectively blocks LPA-induced PAK1 activation and cell migration. Moreover, our results show that PI3K inhibitor can also inhibit LPA-induced ERK activation. Consequently, we conclude that PI3K is an upstream component of the PAK1/ERK signaling pathway in LPA-stimulated breast cancer cells.

It is not clear whether PI3K mediates ERK activation through other ways in our system. Akt is a crucial downstream effector of PI3K and is likely to be responsible for many biological consequences of PI3K activation. Previous studies demonstrated that Ras-Raf-MEK-ERK and PI3K-Akt signaling pathways can crosstalk, and reported that activation of Raf-MEK-ERK pathway by downregulated Akt in some cell types [48]–[50]. Cechin SR *et al*., on the other hand, showed that in glioma cells, LPA induced ERK activity was completely dependent on PI3K, and the PI3K/Akt pathway was not reduced significantly by inhibition of ERK, suggesting that the ERK activity could be implicated in the stimulation of PI3K pathway by LPA [51]. The role of several other signaling molecules that function downstream of PI3K in controlling ERK activation and cell migration remains to be determined. Then another question arises about the relationship between the activation of PI3K and PAK1 after LPA treatment. Although activation of PAK1 by PI3K via sequential activation of Rac and Cdc42 is well characterized [33], [52], a number of GTPase-independent mechanisms may also modify PAK activity and function. For example, PAK1 can be directly phosphorylated on Thr423 by PDK1 [53]. In addition, PI3K might also directly activate PAK1. Previous study has reported that even though Cdc42/Rac1 or Akt are not activated, PI3K activation can still induce PAK1 activity in renal proximal tubule cells, and it also demonstrates that PI3K induces PAK1 activation through binding to the N-terminal regulatory domain of PAK1 (amino acids 67–150) [47]. The mechanism whereby PI3K regulates PAK1 activation in this study remains to be elucidated.

Many studies suggest that ROS-induced damage is associated with aging and various degenerative diseases. Elevated oxidative status has also been found in some types of cancer cells, which contributes to carcinogenesis [54]. Although ROS plays a central role in the key intracellular signal transduction pathway for a variety of cellular processes, a functional significance of PI3K-PAK1-ERK signaling by ROS was not described. The earlier report showed that PDGF-induced migration of vascular smooth muscle cells (VSMC) is ROS-dependent and Src/PDK1/PAK1 signaling pathway is identified as a ROS-sensitive mediator of cell migration [55]. In contrast, our results show that LPA treatment resulted in a significant increase in the production of ROS in breast cancer cell line MDA-MB-231. Furthermore, decreased activation of PI3K and reduced phosphorylation of PAK1 and ERK were observed after inhibition of ROS production, which correlated with ablation of cell migration, thus suggesting a dependency of ROS on PI3K-PAK1-ERK signaling pathway in MDA-MB-231 cells.

It remains unclear how LPA causes the elevation of ROS in breast cancer cells. Previous studies demonstrate that LPA enhances Rac1 activation, and Rac1 has been identified as a necessary component for ROS generation in a variety of cells in response to growth factors and cytokines [56], [57]. It is noteworthy that Rac1 constitutes part of the structure of NADPH oxidase and, in this manner, participates in the control of the intracellular ROS machinery [57]. Therefore, it may be reasonable that LPA stimulates ROS generation in breast cancer cells through the activation of Rac1.

In summary, we have identified a signaling pathway that is implicated in LPA-induced breast cancer cell migration. LPA treatment can lead to the activation of the PI3K/PAK1/ERK cascade in breast cancer cells and contribute to breast cancer migration. We have also found that LPA can stimulate ROS generation in breast cancer cells, which may serve as an important mediator for LPA to stimulate breast cancer cell migration through the activation of PI3K/PAK1/ERK signaling pathway. These findings are of potential pathophysiological importance for understanding the integration of migration-related signaling and shed light on new therapeutic targets for breast cancer.

## Materials and Methods

### Cells and plasmids

Human breast cancer cell line MDA-MB-231 was obtained from the American Type Culture Collection (ATCC, HTB26). Cells were maintained in L-15 medium supplemented with 10% (v/v) fetal bovine serum (FBS), 100 unit penicillin/ml, 100 µg/ml streptomycin and cultured at 37°C in a humidified atmosphere. Cells were grown on coverslips for fluorescence staining and on plastic dishes for protein extraction. Cells were made quiescent by serum starvation overnight followed by drug treatment.

pCMV6M plasmid containing PAK1 K299R mutant insert was kindly provided by Dr. Jonathan Chernoff, and has been described previously [18]. The K299R PAK1 insert was removed from the pCMV6M vector and inserted into the multiple cloning site of the pEGFP-N1 vector. MDA-MB-231 cells were transfected with either the pEGFP-N1 vector or PAK1 K299R expression pEGFP-N1 vector using lipofectamine 2000. Maximal expression was achieved at 48 h post transfection and the transfection efficiency was approximately 40% as detected by green fluorescent protein (GFP) under microscopic observations.

### Reagents and antibodies

Oleoyl-L-α-lysophosphatidic acid sodium salt (LPA), N-acetyl-L-cysteine (NAC), LY294002 were purchased from Sigma, USA. U0126 was obtained from Alexis, Switzerland. Trypsin and Dimethyl Sulfoxide (DMSO) were obtained from Amresco, USA. L-15 medium was the product of Gibco, USA. FBS was purchased from Hyclone, USA. Enhanced Chemiluminescence (ECL) reagent kit was purchased from Pierce, USA. Cell culture inserts were the product of Falcon, USA. The primary antibodies including rabbit anti-Akt antibody, rabbit anti-phospho-Akt (Ser473) antibody, rabbit anti-PAK1 antibody, rabbit anti-PAK1 (Thr423) antibody were purchased from Cell Signaling Technology, USA. Mouse anti-ERK antibody and goat anti-phospho-ERK (Thr202/Tyr204) antibody were obtained from Santa Cruz, USA. Mouse anti-actin antibody was purchased from Chemicon, USA. Corresponding HRP-conjugated secondary antibodies raised in goats were obtained from Santa Cruz, USA. TRIzol reagent, Lipofectamine 2000, 2′,7′-Dichlorofluorescein diacetate (CM_2_-DCFHDA) and dihydroethidium (DHE) were obtained from Invitrogen, USA.

### Wound closure assay *in vitro*

MDA-MB-231 cells were plated in a 96-well plate. Approximately 48 h later, when cells were 95∼100% confluent, cells were incubated overnight in L-15 supplemented with 0.1% (w/v) BSA. Wounding was performed by scraping through the cell monolayer with a 10 µl pipette tip. Medium and nonadherent cells were removed, and cells were washed twice with PBS, and new medium with or without LPA coupled with various inhibitors was added. Cells were permitted to migrate into the area of clearing for 18 h. Wound closure was monitored by visual examination under microscope.

### Transwell migration assay

MDA-MB-231 cells in exponential growth were harvested, washed, and suspended in L-15 medium without FBS. Cells (1×10^6^) were seeded into polycarbonate membrane inserts (8 µm pore size) in 24-transwell cell culture dishes. Cells were allowed to attach to the membrane for 30 min before the addition of inhibitors. The lower chamber was filled with 800 µl of L-15 medium without FBS containing 10 µM LPA as a chemoattractant. Cells were permitted to migrate for 3 h. After the incubation, stationary cells were removed from the upper surface of the membranes. The cells that had migrated to the lower surface were fixed and stained with 0.1% crystal violet. The number of stained cells was counted under an ocular microscope.

### Cell proliferation assays

MDA-MB-231 cells were cultured in the absence or presence of LPA for 24 or 48 h and the proliferation of the cells was analyzed by staining with propidium iodide and flow cytometry analyses or MTT assay as described previously [58].

### Immunofluorescence staining

Cells used for immunostaining were fixed in 3.7% paraformaldehyde in PBS for 30 min, permeabilized in 0.1% Triton X-100 and blocked in PBS containing 1% BSA for 1 h at room temperature. The cells were incubated with anti-phospho-ERK (Thr202/Tyr204) for 2 h followed by incubation with rhodamine-conjugated anti-rabbit antibody for 1 h at room temperature within a moist chamber. After wash with 0.05% Tween 20-PBS, the samples were mounted with aqueous mounting medium. Images were collected using an Olympus BX51 microscope coupled with an Olympus DP70 digital camera.

### Intracellular ROS staining

MDA-MB-231 cells were seeded in 6-well plates at the density of 1×10^5^ cells per well on a cover-slip overnight. Cells were stained with CM_2_-DCFHDA (5 µM) or DHE (10 µM) for 15 min at 37°C, then washed with PBS thrice, and fixed with 4% formaldehyde. Images were captured using an Olympus BX51 microscope coupled with an Olympus DP70 digital camera. Green fluorescence (DCF) intensity was quantitated using the microscope with 485-nm excitation and 530-nm emission settings, respectively. Red fluorescence (HE) fluorescence was measured with 480-nm excitation and 650-nm emission settings.

### SDS-PAGE and western blot analysis

Subconfluent cells were lysed with ice-cold RIPA lysis buffer (50 mM Tris, 150 mM NaCl, 1% Triton X-100, 1% sodium deoxycholate, 0.1% SDS, 1 mM sodium orthovanadate, 1 mM sodium fluoride, 1 mM EDTA, 1 mM PMSF, and 1% cocktail of protease inhibitors) pH 7.4. The lysates were then clarified by centrifugation at 12,000 g for 20 min at 4°C. Protein concentration in the supernatant was quantitated by the protein assay kit (Bio-Rad). Equal amount of samples was run on a 10% sodium dodecyl sulphate polyarcylamide gel electrophoresis (SDS-PAGE). The samples were then transferred onto polyvinylidene difluoride (PVDF) membrane with a Bio-Rad transfer unit at 20 V for 1 h at room temperature. Membranes were blocked in blocking buffer (5% skim milk, 0.05% Tween-20 in Tris-buffered saline) for 60 min followed by incubation with the primary antibodies and then incubated with the corresponding HRP-conjugated secondary antibodies. The immunoblotted proteins were visualized with ECL reagents. Protein expression was normalized against β-actin.

### Reverse transcription polymerase chain reaction (RT-PCR)

Total RNAs were isolated with TRIzol reagent. First-strand cDNAs were synthesized using total RNAs, avian myeloblastosis virus (AMV) reverse transcriptase, and an oligo (dT) primer. Primers used for PCR amplification were as follows: GAPDH: 5′-TGAACGGGAAGCTCACTGG-3′ (sense) and 5′-TCCACCACCCTGTTGCTGTA-3′ (antisense) (307 bp); MMP-9: 5′-TCCCTGGAGACCTGAGAACC-3′ (sense) and 5′-CGGCAAGTCTTCCGAGTAGTT-3′ (antisense) (308 bp); VEGF: 5′-CGGGAACCAGATCTCTCACC-3′ (sense) and 5′-AAAATGGCGAATCCAATTCC-3′ (antisense) (233 bp). The PCR for GAPDH was performed in 26 cycles with the following cycle profile: 95°C for 30 s, 58°C for 30 s, and 72°C for 30 s. The PCR for MMP-9 was set up for 28 cycles with the following cycle profile: 95°C for 30 s, 49°C for 30 s, and 72°C for 40 s. The PCR for VEGF was set up for 28 cycles with the following cycle profile: 95°C for 30 s, 56°C for 30 s, and 72°C for 30 s. The PCR products were resolved by electrophoresis on 1% agarose. Images of electrophoresis were taken using the ChemiDOC XRS Imaging system (BIO-RAD Laboratories, Hercules, CA, USA).

### Statistical analysis

Statistical analyses were carried out using SPSS software. Student's *t* test was used to analyze the differences between two groups. One-way ANOVA followed by SNK tests were employed for multiple paired comparisons. Statistical significance was considered at *P*<0.05.

## Supporting Information

Figure S1 **Dose-dependent effects of LPA on ERK activity.** Serum-starved MDA-MB-231 cells were treated with 0.1, 1, 5 or 10 µM LPA for 15 min. After stimulation, cells were analyzed for P-ERK or total ERK as described under “Experimental Procedures.”Total RNA was collected and analyzed for MMP-9 RNA as described under “Experimental Procedures.” A representative western blot from one of 3 independent experiments shows similar results.
(TIF)

Figure S2 **PI3K and ERK activation is required for LPA-stimulated MMP-9 induction.** Serum-starved MDA-MB-231 cells were treated with 10 µM LPA for the indicated periods. Total RNA was collected and analyzed for MMP-9 mRNA as described under “Experimental Procedures.” After treatment with 10 µM LY294002 or 10 µM U0126 for 30 min, cells were stimulated with 10 µM LPA for 1 h and then cells were analyzed for VEGF and MMP-9 mRNA levels. A representative RT-PCR from one of 3 independent experiments shows similar results.
(TIF)
