# Supplementary figures and images for: Lysophosphatidic Acid Induces MDA-MB-231 Breast Cancer Cells Migration through Activation of PI3K/PAK1/ERK Signaling
Source: PLoS One. 2010 Dec 30;5(12):e15940. doi: 10.1371/journal.pone.0015940 (PMC3012724; doi:10.1371/journal.pone.0015940)

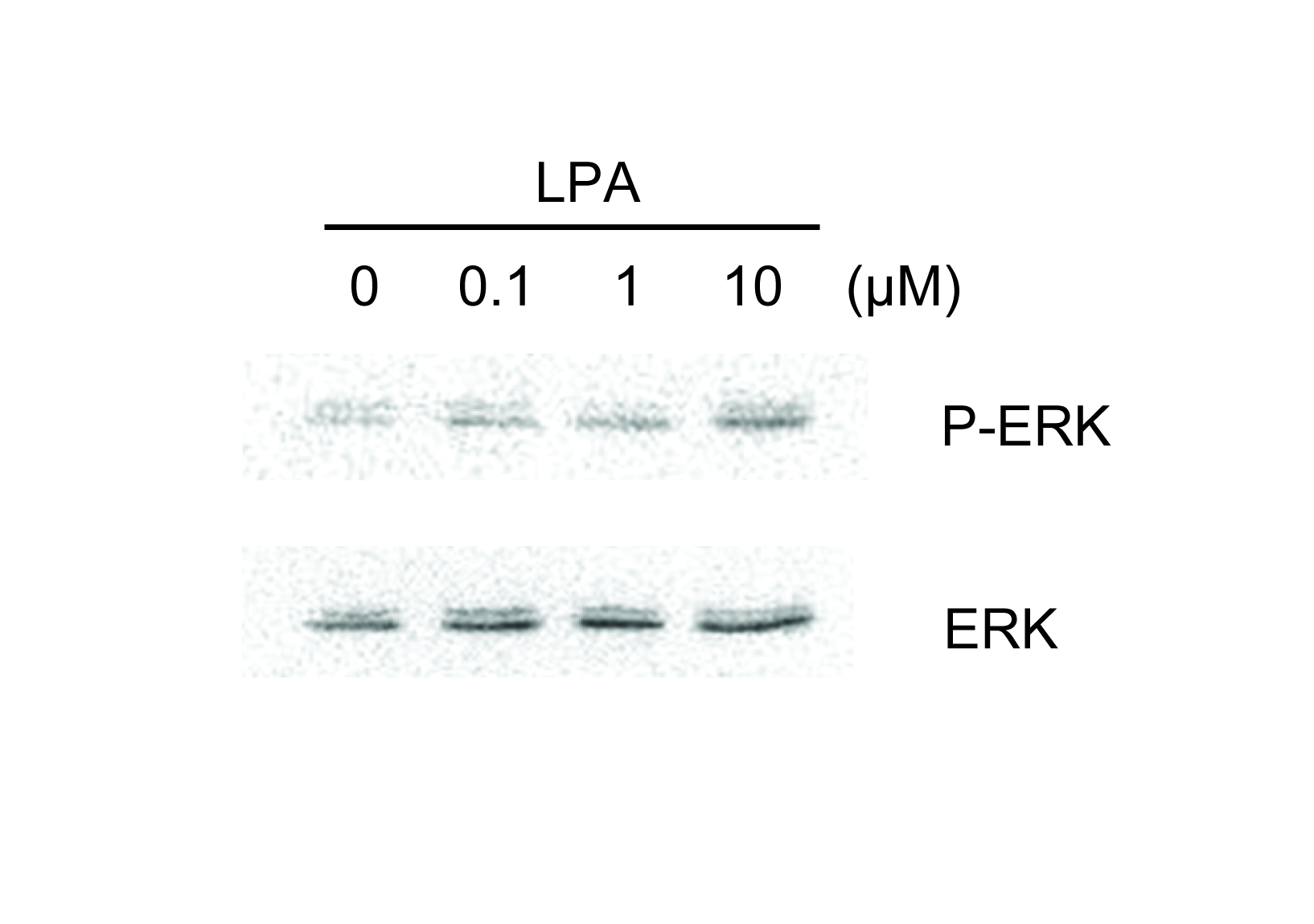

Supplement: Figure S1 — Dose-dependent effects of LPA on ERK activity. Serum-starved MDA-MB-231 cells were treated with 0.1, 1, 5 or 10 µM LPA for 15 min. After stimulation, cells were analyzed for P-ERK or total ERK as described under “Experimental Procedures.”Total RNA was collected and analyzed for MMP-9 RNA as described under “Experimental Procedures.” A representative western blot from one of 3 independent experiments shows similar results. (TIF) [file pone.0015940.s001.tif]

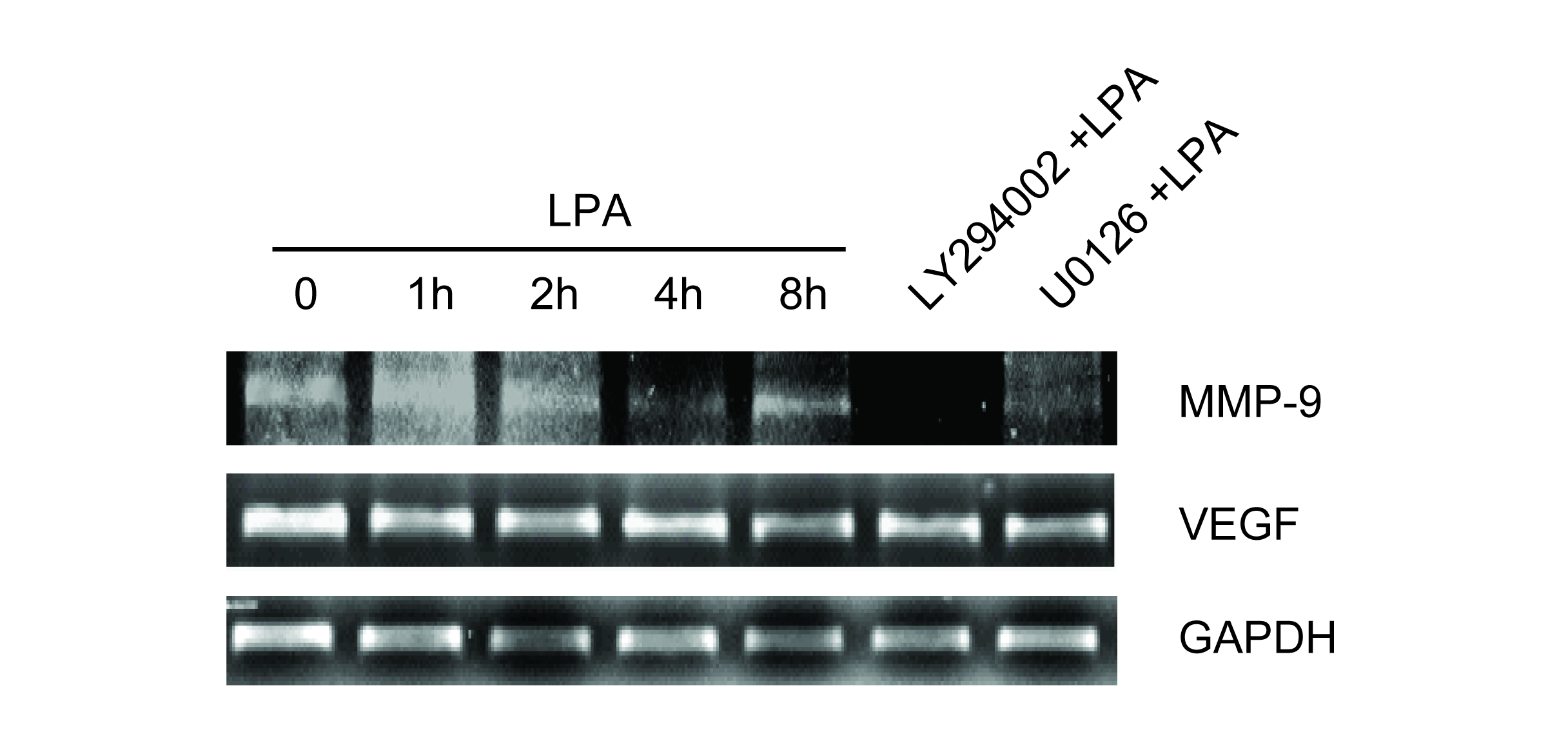

Supplement: Figure S2 — PI3K and ERK activation is required for LPA-stimulated MMP-9 induction. Serum-starved MDA-MB-231 cells were treated with 10 µM LPA for the indicated periods. Total RNA was collected and analyzed for MMP-9 mRNA as described under “Experimental Procedures.” After treatment with 10 µM LY294002 or 10 µM U0126 for 30 min, cells were stimulated with 10 µM LPA for 1 h and then cells were analyzed for VEGF and MMP-9 mRNA levels. A representative RT-PCR from one of 3 independent experiments shows similar results. (TIF) [file pone.0015940.s002.tif]
